# Comparative subcutaneous and submuscular implantation of an electroencephalography device for long term electroencephalographic monitoring in dogs

**DOI:** 10.3389/fvets.2024.1419792

**Published:** 2024-07-12

**Authors:** Casey B. Rogers, Sebastian Meller, Nina Meyerhoff, Holger A. Volk

**Affiliations:** ^1^Department of Small Animal Medicine and Surgery, University of Veterinary Medicine Hannover, Hannover, Germany; ^2^Center for Systems Neuroscience Hannover, Hannover, Germany

**Keywords:** EEG, long-term epilepsy monitoring, continuous EEG, EEG implant, epilepsy

## Abstract

**Background:**

Implantable electroencephalography (EEG) recording devices have been used for ultra-long-term epilepsy monitoring both in clinical and home settings in people. Objective and accurate seizure detection and recording at home could be of great benefit in diagnosis, management and research in canine idiopathic epilepsy (IE). Continuous EEG monitoring would allow accurate detection of seizure patterns, seizure cycles, and seizure frequency. An EEG acquisition system usable in an “out of clinic” setting could improve owner and veterinary compliance for EEG diagnostics and seizure management.

**Objectives:**

Whether a subcutaneous ultra-long term EEG monitoring device designed for humans could be implanted in dogs.

**Animals:**

Cadaver study with 8 medium to large breed dogs.

**Methods:**

Comparatively using a subcutaneous and submuscular approach to implant the UNEEG SubQ-Implant in each dog. Positioning was controlled via CT post implantation and cranial measurements were taken.

**Results:**

In four of the eight dogs a submuscular implantation without any complications was possible. Complications were close contact to the optic nerve in the first approaches, before the implantation angle was changed and in the smallest dog contact of the implant with the orbital fat body. Cranial measurements of less than 95 mm length proved to be too small for reliable implantation via this approach. The subcutaneous approach showed severe limitations and the implant was prone to dislocation.

**Conclusion:**

The UNEEQ SubQ-Implant can be implanted in dogs, via submuscular approach. CT imaging and cranial measurements should be taken prior to implantation.

## Introduction

Canine idiopathic epilepsy (IE) is a brain disease characterized by spontaneous recurrent seizures. It is a common neurological condition, affecting 0.6–0.75% of dogs (1, 2). The current suggested diagnostic approach is divided into three tiers of confidence levels for the diagnosis of IE (3). Tier I and II are based on subjective criteria such as description of episodes, viewing of episodes, physical and neurological examinations, as well as unremarkable advanced tests like blood and cerebrospinal fluid analysis and magnetic resonance imaging (3). For epilepsy management owners are often recommended to keep a “seizure diary.” However, it is discussed that an underreporting of seizure frequency in dogs is commonly given (4). Tier III level of IE diagnosis uses electroencephalography (EEG) for objective confirmation of seizure events. EEG is additionally being used to investigate sleep and cognition in dogs and can aid in diagnosing canine cognitive dysfunction syndrome (CCDS) (5). A syndrome similar to Alzheimer’s disease in people, which can cause clinical signs such as confusion, anxiety, disturbance of the sleep/wake cycle and decreased interaction with owners in dogs (6–8). Recent studies show that fewer than 50% of veterinary neurologists perform EEGs in their diagnostics (9). Limiting factors for the more frequent use being equipment availability, insufficient cases and financial costs to clients (9). Abnormal electric activity is often found in epileptic dogs, without the necessity of an active seizure (10). However, inter-ictal short-term EEGs have a lower diagnostic yield and can have unremarkable findings (11–13), showcasing that an ictal EEG recording highly increases the diagnostic value. With unpredictable seizure onsets, ictal EEG however cannot always be achieved in routine diagnostics, making it more likely to record a seizure event during long term EEG monitoring. Since 2019 implantable EEG recording devices have been used for ultra-long-term epilepsy monitoring both in clinical and home settings in people (14–18). Objective and accurate seizure detection and recording at home could be of great benefit in diagnosis, management and research in IE (19). An EEG acquisition system usable in an “out of clinic” setting could improve owner and veterinary compliance for EEG diagnostics and seizure management, potentially without compromise in diagnostic quality as studies with people have shown (14, 17, 18). Furthermore, continuous EEG monitoring would allow accurate detection of seizure patterns, seizure cycles and seizure frequency (20). In addition, this technology should help resolve the low accuracy of seizure diaries compiled by owners of dogs with IE.

With EEGs often being challenging to perform in a clinical setting (3), this study aims to investigate whether a subcutaneous ultra-long term EEG monitoring device designed for humans could be implanted in dogs.

## Materials and methods

### Device

**Figure 1:**
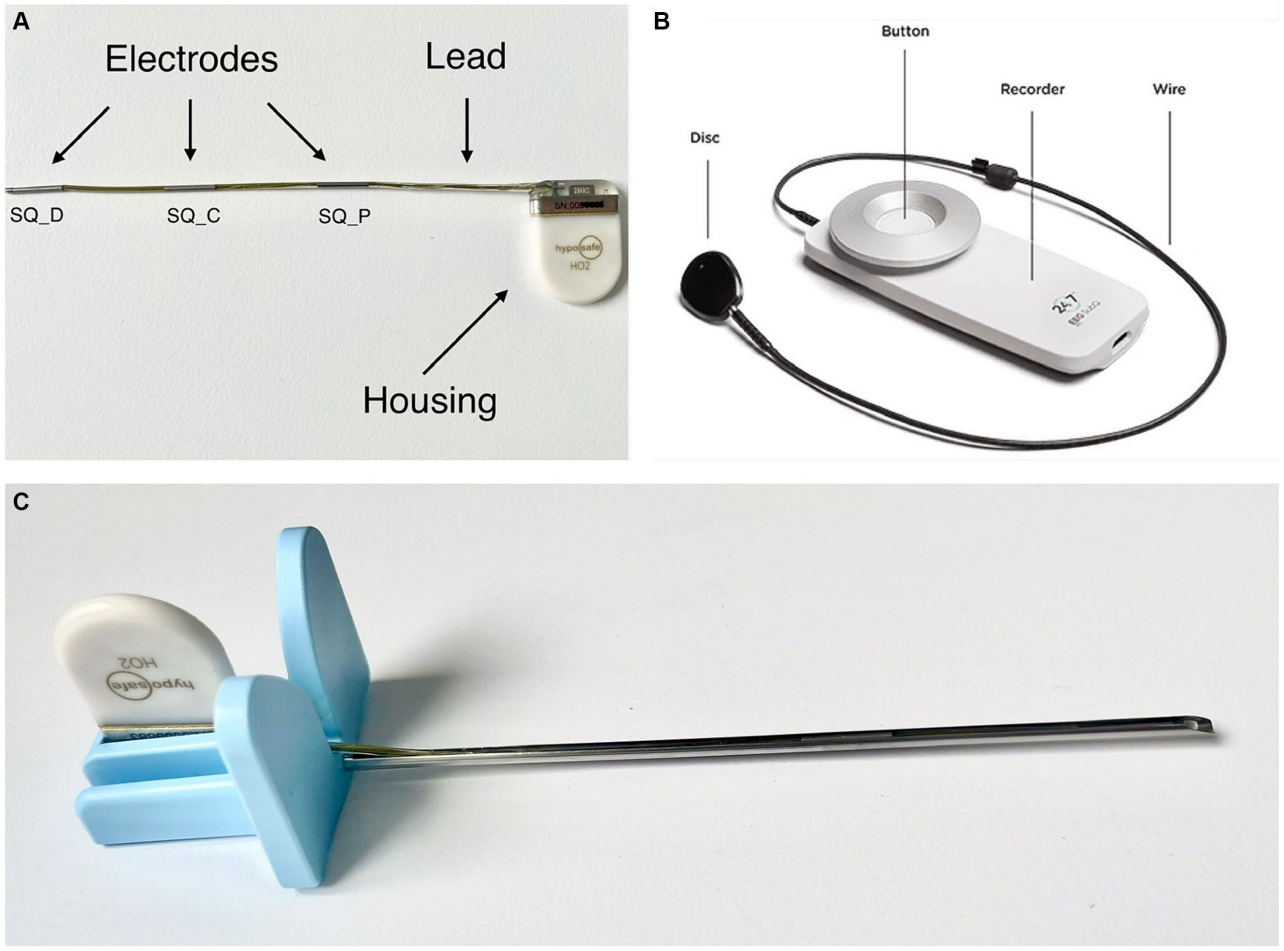
**(A)** UNEEG SubQ-implant. The electrodes are named SQ_P (subcutaneous contact point proximal), SQ_C (subcutaneous contact point central) and SQ_D (subcutaneous contact point distal). **(B)** 24/7 EEG^™^ SubQ recorder, the wearable, external part of the electroencephalography monitoring solution. The disk attaches to the implanted housing. In people it can be attached to clothing via a magnet. Picture source: UNEEG Medical, https://www.uneeg.com/recorder. **(C)** UNEEG Sub-Q introduction needle with inserted implant.

The subcutaneous EEG system (UNEEQ SubQ-Implant) used in this study consists of two components: The implant (Figure 1A) and an external recorder (Figure 1B). In addition, an introducing needle (Figure 1C) is used to aid the implantation procedure. In this study the implantation of said EEG monitoring solution was tested, using the introduction needle for sub-cutaneous or submuscular implantation.

The implant consists of a ceramic housing and a lead with three electrodes and provides two-channel measurements from a single location. The EEG data are measured in μV and split into two channels: “EEG SQ_D-SQ_C”—measured from SQ_C to SQ_D and “EEG SQ_P-SQ_C”—measured from SQ_C to SQ_P (Figure 1A). The housing measures 24 × 17 × 3.3 mm, the lead 103 mm containing three electrodes with an outer diameter of 1.06 mm and a length of 10 mm each.

A small external device (Figure 1B) is inductively coupled to the implanted housing, powering it via this link. The external device receives and stores the EEG signals, storing at least 30 days of data. The data is collected via two channels with 207 Hz and 10-bit sampling. The recorded EEG data can be streamed to a secure cloud environment for storage, analysis, and visual review. The EEG data are analysed by automated seizure detection algorithms, and suspected seizure activity is highlighted for subsequent expert visual review (21). However, this part of the EEG monitoring solution was not in the scope of this study.

### Study design and animals

A cadaver study was performed at the University of Veterinary Medicine Hannover. Eight canine cadavers were used for this study (Table 1). The dogs were donated to science following their euthanasia with written consent by their owners. Due to the size of the device, implantation in dogs ranging from 20 kg to 59 kg was investigated.

**Table 1:** Breed, sex, age, weight in kg, cranial measurements, and reason for euthanasia of the dogs used in this study.

	Breed	Sex	Age (years)	Weight (kg)	Cranium in mm (length × height)	Reason for euthanasia
Dog 1	Labrador Retriever	F	10	33	103 × 70	Adrenal neoplasia
Dog 2	Labrador Retriever	M	14	32	108 × 72	Cardiac disease
Dog 3	Mixed breed	M	6	32	108 × 65	Pneumothorax
Dog 4	Long Haired Collie	M	2	20	95 × 59	Sepsis
Dog 5	German Sheperd Dog	Fs	4	34	109 × 66	Gastric torsion
Dog 6	Bernese Mountain Dog	Fs	8	34	105 × 67	Urethral neoplasia
Dog 7	Elo	Fs	13	23	90 × 58	Neoplasia
Dog 8	Bordeaux Mastiff	M	5	59	125 × 86	Cardiac disease

Dogs were used in the order 1–8. M, male; F, female; Fs, female, spayed.

### Implantation and control of positioning

In each dog two implantation approaches were performed. First, the implant was introduced subcutaneously along the zygomatic arch (subcutaneous approach). Second, on the other side a submuscular approach similar to the surgery described for people in a publication by Djurhuus et al. (16) was additionally examined. A computed tomography (CT)^1^ scan was performed following both subcutaneous and submuscular implantation to evaluate the position of the implant. The CT scans included soft tissue and bone windows with a metal artefact reduction filter. The cranium of each dog was measured using the CT images. At midline, noting the length from the occiput to the level of the zygomatic process of the frontal bones and the height from the basisphenoid bone in a 90°C angle to the sagittal suture.

### Subcutaneous approach

1. On the shaved head, a linear, vertical incision of about 25 mm length was made just above the caudal border of the zygomatic arch and about 10 mm caudal to the planned caudal border of the housing.
2. A subcutaneous pocket for the housing was created.
3. The implant was fitted in the introducing needle.
4. The introducing needle can be bent carefully to fit the curvature of the patient’s head.
5. Using the introducing needle the implant was slowly inserted into the subcutaneous tissue. Following the curvature of the zygomatic arch the introducing needle was embedded until the tip reached just past the anterior border of the zygomatic arch, in distance to the eye and auriculopalpebral nerve.
6. Fixating the lead with forceps, the introducing needle was withdrawn carefully leaving the lead *in situ*.
7. The housing was inserted into the subcutaneous pocket and the skin closed with surgical sutures.

### Submuscular approach

1. On the shaved head, a linear, vertical incision of about 25 mm length was made above the masseter in the occipital area, midline between occiput and ear base and about 10 mm behind the planned caudal border of the housing (Figure 2).

**Figure 2:**
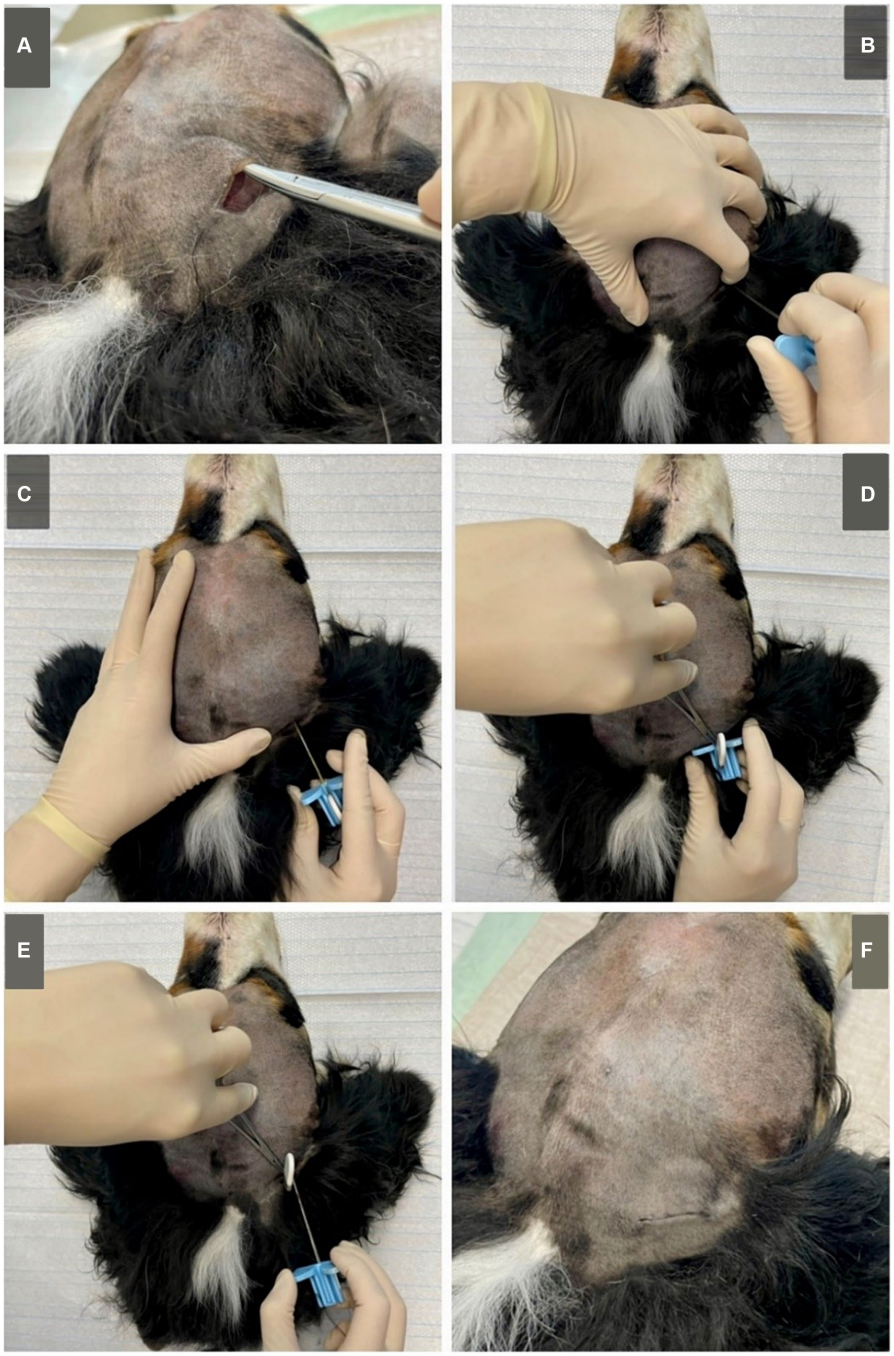
Submuscular approach on a Bernese Mountain Dog (cadaver, dog 6). **(A)** A subcutaneous pocket is created to fit the housing of the implant. **(B)** The implant is vertically inserted through the masseter, at about 1/3 of the distance between the caudal part of the zygomatic arch and the occiput using the introduction needle. When reaching the skull, **(C)** the introduction needle is orientated horizontally and angled towards the zygomatic process of the frontal bone. It is then inserted, following the skull. **(D)** When fully inserted (or in smaller dogs inserted to the desired length) the lead can be fixated using forceps. **(E)** The introduction needle is carefully removed, keeping the implant fixated in place. **(F)** The implant is fitted in the subcutaneous pocket and the skin is closed using surgical sutures (pictured here, intracutaneous stiches). Although not pictured, when applying to live animals, a sterile approach is needed. Standard surgical preparation including shaving, washing, disinfection of the skin and sterile covering of the surgical area are required.
2. A subcutaneous pocket for the housing was created.
3. The implant was fitted in the introducing needle.
4. To help orientation, the thumb can be placed on the occiput and the middle finger on the caudal edge of the zygomatic arch. Placing the index finger at the ear base, approximately at 1/3 of the length between the caudal aspect of the zygomatic arch and the occiput. Using the other hand, the introducing needle and implant were vertically inserted through the masseter until the tip reaches the skull.
5. The introduction needle was then angled horizontally and aimed towards the zygomatic process of the frontal bone/medial cantus of the eye. Following the skull, it was embedded underneath the masseter, until the implant was fully inserted and only the housing remains above the masseter (or in smaller dogs inserted to the desired length).
6. Fixating the lead with forceps, the introducing needle was withdrawn carefully leaving the lead *in situ*.
7. The housing was inserted into the subcutaneous pocket and the skin is closed with surgical sutures.

## Results

The cranial measurements for each dog can be found in Table 1.

### Subcutaneous approach

**Figure 3:**
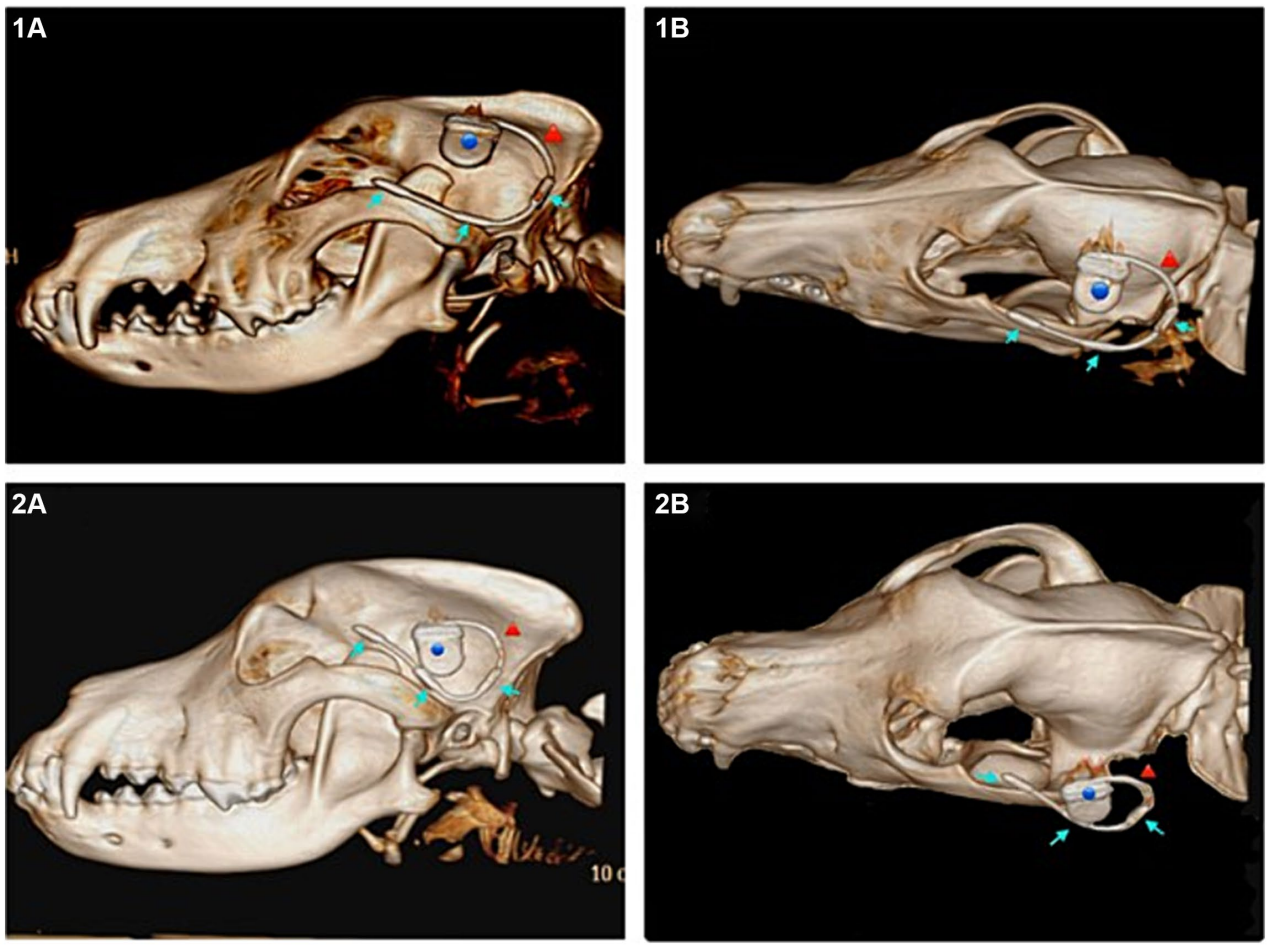
Subcutaneous approach. **(1A)** Lateral view of the subcutaneous implant in a 3D reconstruction of the computed tomography (CT) images of dog 4, ideal positioning. **(1B)** Dorsolateral view of the same dog (dog 4). **(2A)** Lateral view of the dislocated subcutaneous implant in a 3D reconstruction of the CT images of dog 6. **(2B)** Dorsolateral view of the same (dog 6). Blue dot marking the housing, red arrow marking the lead, blue arrows marking the three electrodes of the implant.

A good placement result was achieved in five of the eight dogs (dogs two, four, five, seven, eight) (*n* = 5/8). With the implant being positioned along the zygomatic arch (Figure 3) and the electrodes overlying the frontal, temporal and occipital lobes. In the remaining three dogs the electrode was, in the cases of dogs one and six, dislocated in the area of the rostral electrode with it being diverted dorsally (*n* = 2/3) (example Figure 3). And in dog three the entire lead dislocated in the subcutaneous tissue (*n* = 1/3). Regardless of the final position all implants were prone to dislocation when the tissue, especially the skin was subjected to movement.

### Submuscular approach

**Figure 4:**
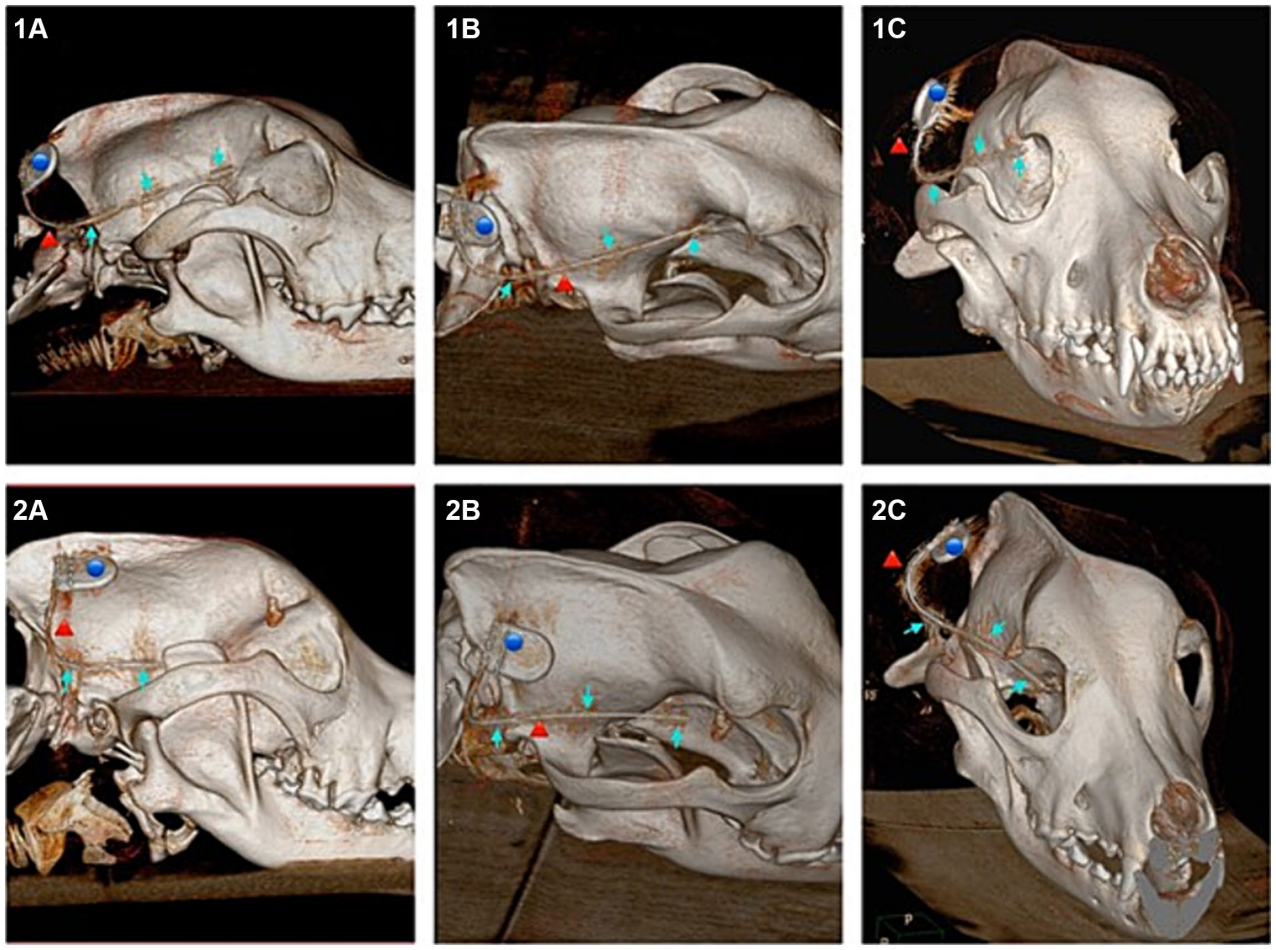
Showing 3D reconstructions of the skull from computed tomography (CT) images after submuscular implantation. Dog 6 (images numbered 1) with ideal implantation and dog 1 (images numbered 2) implanted with probable contact to the optic nerve. **(1A)** Lateral view of dog 6. **(1B)** Dorsolateral view of dog 6. **(1C)** Rostral view of dog 6. **(2A)** Lateral view of dog 1. **(2B)** Dorsolateral view of dog 1. **(2C)** Rostral view of dog 1. Blue dot marking the housing, red arrow marking the lead, blue arrow marking the three electrodes of the implant.

**Figure 5:**
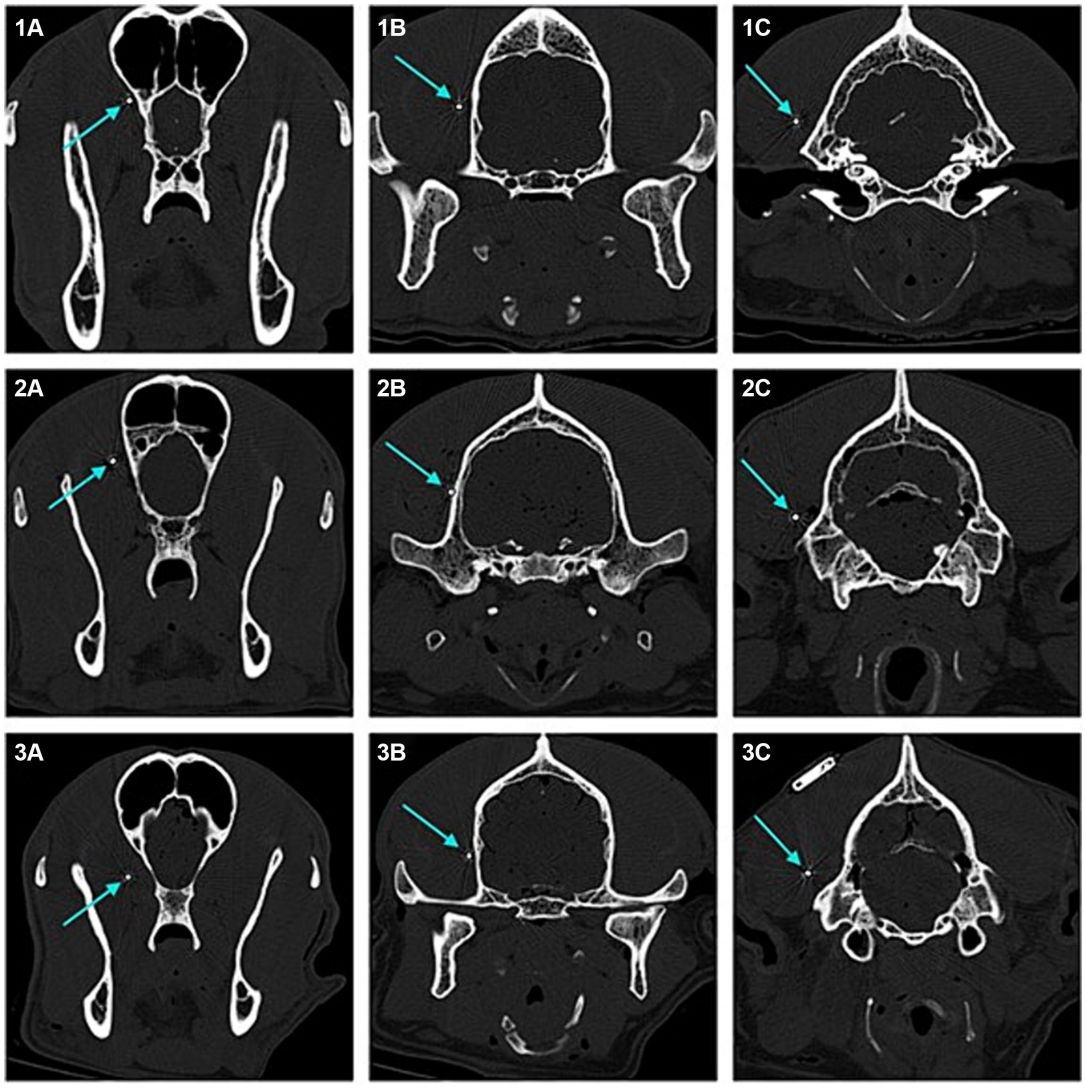
Transverse 2D computed tomography (CT) images (bone window, metal artefact reduction) of the dogs’ heads taken at the position of each of the three electrodes of the implant (submuscular implantation). The electrodes are pointed at with blue arrows. Images numbered 1 show the positions for dog 5, with good implant position. Images numbered 2 show the positions for dog 6, with good implant position. Images numbered 3 show the positions for dog 1, with the rostral laying electrode close to the optic nerve. **(1A)** Rostral laying electrode of the implant located at the level of the frontal lobe in dog 5. **(1B)** Middle electrode located at the level of the temporal lobe in dog 5. **(1C)** Caudal laying electrode located at the level of the occipital lobe in dog 5. **(2A)** Rostral laying electrode of the implant located at the level of the frontal lobe in dog 6. **(2B)** Middle electrode located at the level of the temporal lobe in dog 6. **(2C)** Caudal laying electrode located at the level of the occipital lobe in dog 6. **(3A)** Rostral laying electrode of the implant located at the level of the frontal lobe, in the area of the optic nerve, in dog 1. **(3B)** Middle electrode located at the level of the temporal lobe in dog 1. **(3C)** Caudal laying electrode located at the level of the occipital lobe in dog 1.

In four of the eight dogs (*n* = 4/8) an implantation without any complications was possible. In the first two dogs (dogs one and two), when angling towards the medial canthus of the eye the implantation resulted in the implant laying in close contact with, or vicinity of the optic nerve. The more caudal aspects of the implant however showed narrow distance to the cranium and good alignment with the temporal and occipital lobes (Figures 4, 5).

In the following dogs the implant was angled towards the zygomatic process of the frontal bone, achieving good placement results and enough distance to structures such as the optic nerve and ophthalmic veins. Dogs three, four, five and six showed good alignment of the electrodes with the occipital lobe, temporal lobe and frontal lobe (Figures 4, 5).

In dog seven a full insertion of the implant resulted in the rostral part of the implant being embedded in the orbital fat body. In dog eight the rostral electrode only reached up to the temporal lobe.

## Discussion

This article represents the first approach to implanting a subcutaneous ultra-long term EEG monitoring device designed for humans in dogs. It was shown that an implantation is possible and in half of the tested dogs, in the submuscular approach a good result in terms of implant position was achieved. Changing the insertion angle towards the zygomatic process of the frontal bone achieved better results in terms of positioning in further distance to the optic nerve and related blood vessels. Whether a penetration of the optic nerve with the implant occurred in the first two dogs during the submuscular approach could not be ruled out. Nevertheless, even close contact to the optic nerve could cause irritation, inflammation and discomfort if applied in patients. The authors therefore recommend positioning the electrode as demonstrated in dogs three to six (Figures 2, 4). However post operative imaging should nevertheless be done, if applied to patients, to identify potential complications like haemorrhage and to ensure a good positioning of the lead. In dog seven the implant reached into the orbital fat body once fully inserted. This dog had the smallest cranial measurements out of the eight dogs used in this study. Despite the cranium of dog four only being 5 mm longer and 1 mm higher than dog seven’s, enough distance to the orbital structures could be seen in dog four. Dog eight proved to have the largest cranium, being a large breed brachycephalic dog. With a length and height difference of almost 30 mm compared to dog four. These measurements did not account for the thickness of the masseter, which also plays a role in implant positioning, since the lead first must be inserted through the entire muscle. Depending on the thickness a large proportion of the lead remains in the muscular tissue, before the desired position is reached. The housing has to remain subcutaneously to be connected with the recorder. Dog eight therefore showed the opposite to dog four, with the head being too large for the electrodes to span over the occipital, temporal and frontal lobe. In cases like this a decision as to which projection areas are desired should be made prior to implantation. This showcases that the size of the dog and measurement of the cranium are crucial for future patient selection. In the dogs investigated, we found that a length of less than 95 mm spanning at midline, from the occiput to the level of the zygomatic process of the frontal bones is too short for implantation via this submuscular approach, similar to the one used in people (16). When implanting this device, but especially in smaller dogs, the positioning should be planned via imaging prior to implantation. This would allow detailed measurements of the cranium to investigate whether the implant can be fitted. The implant leaves little leeway as to the degree of insertion, if all three electrodes are implanted. The use of a neuronavigation system or more invasive surgical procedures involving incising the masseter may allow implantation in smaller dogs.

Whether this implant achieves good resolution and evaluable EEG recordings in dogs was not in the scope of the current study. However, since scalp EEG has relatively low sensitivity for the detection of seizure onsets due to the large distance from the cortex to the surface (22), better results in seizure detection may be possible with this device. The distance to the cortex is reduced immensely in the submuscular approach compared to scalp EEG. The submuscular approach also eliminates a few of the barriers (skin, muscle) that interfere significantly with the recording of the EEG. Since the electrodes are still in contact with the muscles, additional EMG recordings may be required when applying this device to dogs. When using the subcutaneous approach merely the skin is eliminated as a barrier. The subcutaneous approach showed severe limitations in this study. The implant was prone to dislocation when the tissue, especially the skin, was subjected to movement. Thus, despite good positioning results in 62% of these cases, in live dogs, it cannot be guaranteed that the implant would remain in such a position once the patient is awake, and the implant is subjected to movement of the skin, muscles and jaw or ears. When dislocating laterally, over the zygomatic arch, an additional insulating barrier (bone) would be added, decreasing the resolution of the EEG further. The authors therefore cannot recommend the application of a subcutaneous approach in canine patients.

Due to the invasiveness of the procedure, if applied to canine patients, an implantation under local anaesthetic, as used in people (16) would not be possible. To guarantee a secure implantation, without damaging of nerves or blood vessels, a full restraint of the patient would be necessary. For this, a deep sedation with local anaesthesia, or better a general anaesthesia is required. In people side effects such as itching, soreness, or tightness/irritation around the implant and headaches up until 21 days post-surgery, as well as wound infections and skin penetration in the area of the housing were observed (16). Close monitoring, wound and pain management, if applied to canine patients should therefore be ensured. Contraindications named for people, which could be adapted to canine patients include patients at high risk of surgical complications, such as active systemic infection and haemorrhagic disease. Patients with owners who are unable (i.e., mentally or physically impaired) or do not have the necessary assistance to properly operate the device system. Patients who have an infection at the site of device implantation. Patients who require MRI scans following the implantation (23).

This study did not evaluate the connectivity of the recorder to the implanted device in dog. In people the positioning of the housing is recommended to be behind the ear, regardless of the lead position (16). Named reasons for this are that “the housing rests on a relatively flat and stable part of the cranium close to, but preferably outside, the hairline. This position eases the device management and may make the need of regular shaving unnecessary” (16). This can lead to the assumption that dogs equipped with the implant may require regular clipping of the fur surrounding the implant, to guarantee connectivity with the external recorder.

Applications of this device in dogs would allow for continuous EEG recording in dogs over an extended period, ranging from days to months. Continuous EEG plays a crucial role in the diagnosis and management of various neurological conditions, including epilepsy (24, 25). Unlike conventional EEG, which captures brief snapshots of brain activity during short recording sessions, continuous EEG allows for the uninterrupted monitoring of brain electrical activity, during the inter-, pre-, postal, and ictal phases. This offers several important advantages from both a diagnostic and translational standpoint. It provides insights into the dynamics of epileptic activity, enabling clinicians to characterize seizure patterns, frequency, and duration more accurately (26–28). This detailed information can be used for optimizing treatment strategies, including the selection and adjustment of antiseizure medication and seizure forecasting (29, 30). In human medicine, studies have shown that the system used in this study allowed successful seizure forecasting (18, 31). Furthermore, continuous EEG monitoring facilitates the identification of inter-ictal epileptiform discharges (IEDs), which are abnormal electrical signals in the brain that occur between seizures (26, 32, 33). They yield information about the distribution of epileptic events and can be more prevalent than seizures and occur more regularly (26). Detection of IEDs helps confirming the diagnosis of epilepsy and assessing treatment response (34). However, using only a two-channel electrode system may limit the spatial resolution and complexity of the EEG data and may provide less clinically meaningful information than a standard multi-channel EEG, which can help localise the origin of abnormal EEG data, especially when applied with the help of a neuronavigation system (38). The current system will be mainly useful to monitor seizure activity. In an ideal setting, a multichannel EEG system will be used to map epileptic seizure activity to a specific brain area and the placement of the two-lead system will be tailored accordingly. Studies in people have shown that the hippocampus, amygdala, frontal cortex, temporal cortex, and olfactory cortex are the common areas involved in seizures (39). With epileptic foci such as changes within the hippocampus (40) or white matter (41) still being an ongoing topic of research in veterinary medicine as well as seizure induce changes within the brain (42, 43) this system may aid in further research by supplying continuous long-term EEG data. Especially, when implanted to record the anatomical location of interest. Though the system investigated in this study may require a bilateral implantation of the device, using two implants for a good assessment or comparison of both hemispheres. Considering the invasive surgical procedure, potentially involved in implanting one or two devices in dogs, it is essential that the clinical benefits outweigh the risks. The results from human medicine however show promising results that this could also be possible in canine patients (18, 31). The application of this device could have significant clinical benefits regarding disease monitoring, especially in patients where an epileptic focus was previously diagnosed and the device implanted to fit this location accordingly.

From a translational standpoint, continuous EEG recording in dogs holds potential for advancing our understanding of epilepsy pathophysiology and treatment. Dogs with naturally occurring epilepsy share many similarities with human patients in terms of seizure presentation, underlying mechanisms, and treatment response (35–37, 44) Moreover, continuous EEG monitoring in dogs can serve as a valuable preclinical model for testing new antiseizure medication and investigational therapies. The ability to assess drug efficacy, safety, and tolerability in a naturally occurring disease setting in dogs can provide valuable insights that may ultimately benefit human patients with epilepsy (35–37, 45).

This study shows promising results, that subcutaneous ultra-long term EEG monitoring device designed for humans can be implanted in dogs and may qualify for future research outside of this cadaver study. Future uses could cover clinical aspects for epilepsy diagnostics, epilepsy monitoring and seizure forecasting, monitoring responses to antiseizure medication, as well as the use in research for sleep and CCDS or pharmaceutical research.

## Data availability statement

The original contributions presented in the study are included in the article/supplementary material, further inquiries can be directed to the corresponding author.

## Ethics statement

The animal studies were approved by the Doctoral Committee of the University of Veterinary Medicine Hannover, acting as the Ethics Committee, in accordance to the ethics guidelines for cadaver studies. The studies were conducted in accordance with the local legislation and institutional requirements. Written informed consent was obtained from the owners for the participation of their animals in this study.

## Author contributions

CR: Writing – original draft, Writing – review & editing. SM: Writing – original draft, Writing – review & editing. NM: Writing – original draft, Writing – review & editing. HV: Writing – original draft, Writing – review & editing.

## Footnotes

^1^Philips IQon Spectral CT (Philipps Medical Systems GmbH, Hamburg, Germany).
